# Editorial: Neuronal Pathways Affecting Glial Function

**DOI:** 10.3389/fncel.2021.686796

**Published:** 2021-05-07

**Authors:** Yossi Buskila, Erika Gyengési, John W. Morley

**Affiliations:** School of Medicine, Western Sydney University, Penrith, NSW, Australia

**Keywords:** glia, neuron, neurodegenaration, inflammation, dementia

The brain is a neuron-glia system, in which neuronal activity affects glia function and in return, glial cells impact neuronal activity. It is well-established that glia expresses a plethora of ionotropic and metabotropic receptors, and that under physiological conditions they can react to neuronal activity via different pathways, including enhancement of synaptic release of neurotransmitters (Buskila and Amitai, 2010), the release of neurotrophic factors (Liddelow et al., 2017) and Ca^2+^ dependent release of gliotransmitters (Pirttimaki et al., 2017). Moreover, damage associated molecular patterns (DAMP's) released from neurons during pathophysiological conditions can lead to glial reactivity and induction of neuroinflammation (Cunningham et al., 2019). However, the specific pathways and mechanisms by which neurons impact the functional activity of glial cells are still unclear.

In this Research Topic we present a collection of articles discussing some of the pathways and mechanisms by which neurons impact the functional activity of glia during both physiological and pathological conditions.

Stevenson et al. review the recent literature about neuron to glia communication pathways, focusing on studies concerning the impact of neurons on glial activity during neurodegenerative disorders. The authors specifically discuss the overall lack of glial support, due to the dysregulation of neuron to glia interactions, as a key contribution to the etiology and disease progression of neurodegeneration. Following an in depth description of the current standing, including processes by which neuronal signals are amplified via neuromodulation of glial activity and the glial processes that are affected during neurodegeneration, the authors discuss a new therapeutic strategy which place glia as the therapeutic target.

Greferath et al. describes the glial changes in the retina during retinitis pigmentosa. Their findings indicate morphological changes between the dorsal and ventral regions of the retina, and point out the negative correlation between the presence of glia within the subretinal space and the thickness of debris which affects the loss of photoreceptors. They concluded that the breakdown in the outer limiting membrane in the ventral retina is critical for the extension of glial processes and migration of microglia into the subretinal space, that ultimately exacerbates photoreceptor death via release of cytokines (e.g., Ccl5) in this region of the retina.

Pacholko et al. discuss the role astrocytes play in extending and amplifying neuromodulatory actions. Neuromodulators influence transitions between brain states, however these state changes are dynamic and occur over widespread regions of the CNS. Since a simple diffusion of neuromodulators alone is not sufficient to enable such a widespread effect, the authors hypothesize that astrocytes provide the means by which neuromodulators have their widespread influence. From their review of the literature the authors conclude that the interconnected astrocytic syncytium is essential for eliciting and extending neuromodulator effects by promoting synchronicity of neuronal populations associated with the different brain states.

The cholinergic pathways in the CNS are involved in cognitive function and, under normal conditions, are supported by the intimate relationship between cholinergic neurons and glia. This relationship is essential to maintain protection against acute inflammatory and oxidative stresses. Cognitive decline in aging is in some cases associated with pathology, such as in Alzheimer's disease and Lewy Body dementia, but can also occur during physiological aging, and where there is a disruption in the central cholinergic pathways possibly associated with chronic neuroinflammation. Gamage et al. provide a summary of the role of glia, in particular microglia and astrocytes, in protecting against inflammatory and oxidative stress. They provide evidence implicating nicotinic acetylcholine receptors in that role, and that targeting these receptors could provide a therapeutic advantage in the treatment of age-related neurodegenerative diseases and chronic inflammation.

Taking these results and reviews together, it is clear that our understanding of the role of glial cells, including microglia, astroglia and oligodendrocytes, has come a long way from being considered as merely a non-functional glue for neurons, to now being appreciated as a crucial player in supporting neuronal function. While decades of research is now trying to catch up on revealing the true depth and importance of their function and support during both physiological and pathological conditions, what we know today is most likely just still the tip of what could be a very large iceberg. By using modern experimental techniques and genetic manipulations, we will gain more insight into the functional relationship of glia and specific neuronal populations in the central nervous system during both healthy aging and disease conditions. This Research Topic is a good starting point and shows some of the reasons for the exciting promise of future research to reveal more about the function of glial cells.

## Author Contributions

All authors conceived the project, revised the literature, wrote, and approved the manuscript.

## Conflict of Interest

The authors declare that the research was conducted in the absence of any commercial or financial relationships that could be construed as a potential conflict of interest.
